# Developing a low-cost and accessible COVID-19 vaccine for global health

**DOI:** 10.1371/journal.pntd.0008548

**Published:** 2020-07-29

**Authors:** Peter J. Hotez, Maria Elena Bottazzi

**Affiliations:** 1 Texas Children’s Center for Vaccine Development, Departments of Pediatrics and Molecular Virology and Microbiology, National School of Tropical Medicine, Baylor College of Medicine, Houston, Texas, United States of America; 2 Department of Biology, Baylor University, Waco, Texas, United States of America; 3 Hagler Institute for Advanced Study at Texas A&M University, and Scowcroft Institute of International Affairs, Bush School of Government and Public Service, Texas A&M University, College Station, Texas, United States of America; 4 James A. Baker III Institute of Public Policy, Rice University, Houston, Texas, United States of America; WRAIR, UNITED STATES

## Overview

There is an urgent need to advance safe and affordable COVID-19 vaccines for low- and middle-income countries of Asia, Africa, and Latin America. Such vaccines rely on proven technologies such as recombinant protein–based vaccines to facilitate its transfer for emerging market vaccine manufacturers. Our group is developing a two-pronged approach to advance recombinant protein–based vaccines to prevent COVID-19 caused by SARS-CoV-2 and other coronavirus infections. One vaccine is based on a yeast-derived (*Pichia pastoris*) recombinant protein comprised of the receptor-binding domain (RBD) of the SARS-CoV formulated on alum and referred to as the CoV RBD219-N1 Vaccine. Potentially, this vaccine could be used as a heterologous vaccine against COVID-19. A second vaccine specific for COVID-19 is also being advanced using the corresponding RBD of SARS-CoV-2. The first antigen has already undergone current Good Manufacturing Practices (cGMP) manufacture and is therefore “shovel ready” for advancing into clinical trials, following vialing and required Good Laboratory Practice (GLP) toxicology testing. Evidence for its potential efficacy to cross-protect against SARS-CoV-2 includes cross-neutralization and binding studies using polyclonal and monoclonal antibodies. Evidence in support of its safety profile include our internal assessments in a mouse challenge model using a lethal mouse-adapted SARS strain, which shows that SARS-CoV RBD219-N1 (when adsorbed to aluminum hydroxide) does not elicit eosinophilic lung pathology. Together, these findings suggest that recombinant protein–based vaccines based on the RBD warrant further development to prevent SARS, COVID-19, or other coronaviruses of pandemic potential.

*“The thing we have to think about now that’s different is*, *how do we produce vaccines specifically for the developing world if this is a truly global epidemic*.*”—Seth Berkley*, *CEO*, *Gavi*

## Introduction: Disease burden in low- and middle-income countries

As of June 2020, COVID-19 caused by the SARS-CoV-2 coronavirus has infected more than 7 million people globally (confirmed cases) and caused almost 400,000 deaths [1]. Although the epidemic began in China, Europe, and the United States, there are significant concerns about the risks of disease emergence in low- and middle-income nations. There are now more almost 750,000 cases in Brazil, 300,000 cases in India, and 50,000 cases in South Africa, such that COVID-19 will become widespread among the poor living in the group of 20 nations [1, 2]. Moreover, SARS-CoV-2 infection is expected to emerge in the Global South [3]. In the African region of the World Health Organization (WHO), COVID-19 is now spreading in the populated areas of Ghana, Nigeria, and Democratic Republic of Congo, and presumably across the region [1]. In nations such as India, for example, the feasibility of enforcing social distancing in large and crowded urban centers will be particularly daunting [3], so that ensuring access to a safe and affordable COVID-19 vaccine will become a global priority. Dr. Seth Berkley, the CEO of Gavi, the Vaccine Alliance, has highlighted the importance of prioritizing a COVID-19 vaccine specifically for these countries [4].

## Rationale and approach

At least a dozen COVID-19 candidate vaccines are under development using different technology platforms [5], with an emphasis on speed, maximizing safety, and avoiding vaccine-induced immunopathology [6]. Many of these will enlist cutting-edge nucleic acid delivery technologies and other innovative approaches. In the meantime, there is urgency to address and rapidly respond to Gavi’s charge and pursue safe, low-cost, easily administered, and rapidly scalable approaches. For instance, Texas Children’s Center for Vaccine Development (CVD) at Baylor College of Medicine, in collaboration with its nonprofit product development partners—Seattle-based PATH and Infectious Disease Research Institute (IDRI)—have been spearheading a coronavirus vaccine program focusing on recombinant subunit protein vaccines produced in a globally available microbial fermentation platform, and optimized to maximize yield following expression and protein purification [7, 8].

Towards this goal, we are now also developing the SARS-CoV-2 RBD recombinant protein as a potential vaccine candidate, in parallel with the existing CoV RBD219-N1 candidate vaccine, which was previously developed and manufactured under cGMP in 2016 [7–10]. The bulk drug substance has been stored frozen (−70°C to 80°C) and remains stable through ongoing testing. Furthermore, an independent quality assessment confirmed the suitability of the material through Phase 2 clinical trials.

**Fig 1:**
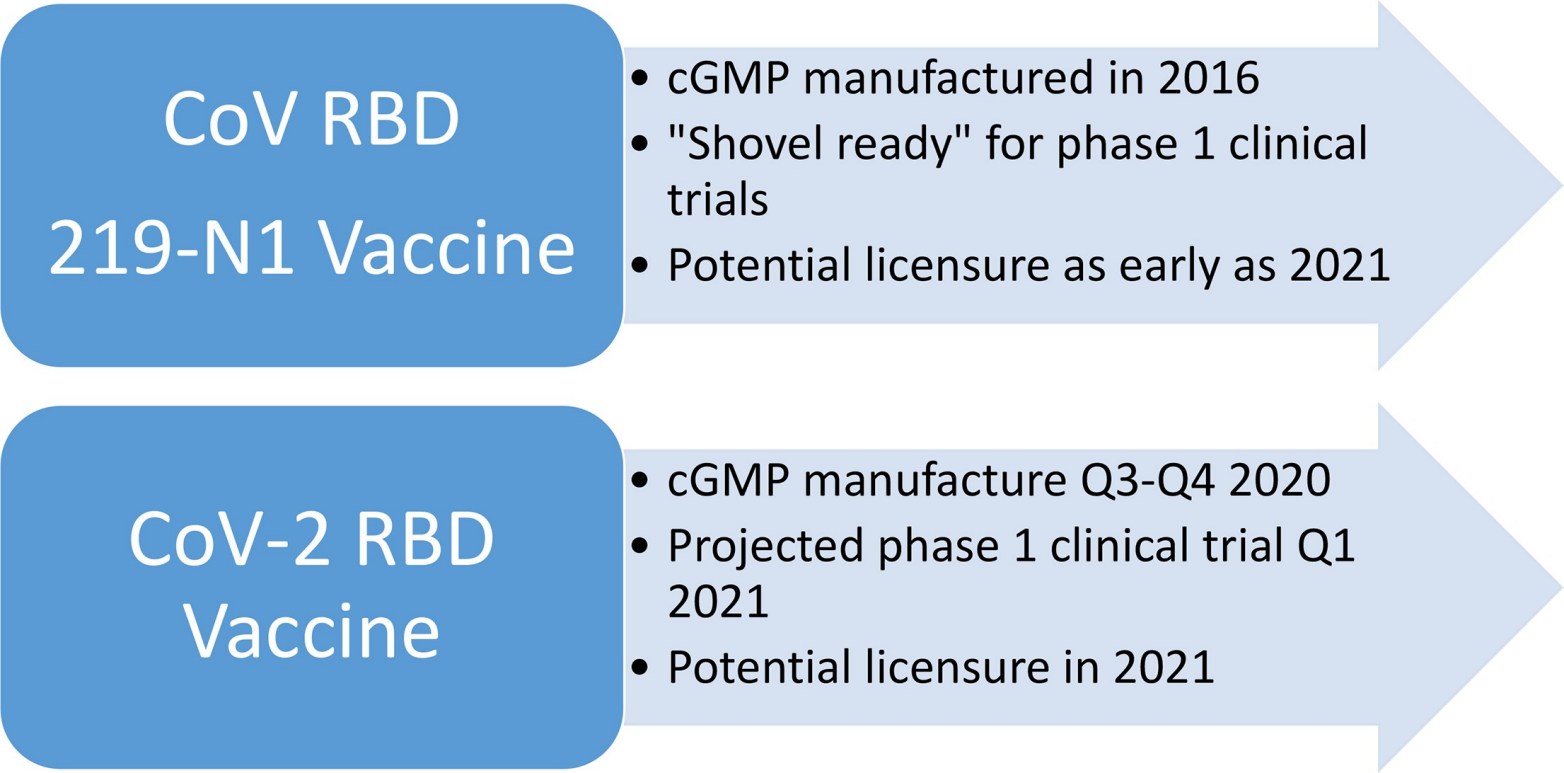
Estimated timelines of coronavirus RBDs as COVID-19 vaccines. cGMP, current good manufacturing practices; RBD, receptor-binding domain.

Both RBD vaccine candidates have potential as vaccine antigens to prevent SARS-CoV-2 infection and/or COVID-19. Overall, our initial approach relies on advancing the already manufactured CoV RBD219-N1 as a heterologous recombinant subunit vaccine to protect against both SARS and COVID-19 [9], and in parallel accelerate the advancement of the SARS-CoV-2 RBD candidate as a homologous COVID-19 vaccine (Fig 1). Our preliminary studies now show that the SARS-CoV-2 RBD candidate, which is specific for the sequence of the SARS-CoV-2, can also be highly produced in the yeast *P*. *pastoris*. Both approaches reinforce each other, as the processes developed for the CoV RBD219-N1 candidate also apply to the SARS-CoV-2 candidate, and both antigens downstream could be further developed as potentially a bivalent or a universal coronavirus vaccine.

The SARS-CoV protein known as CoV RBD219-N1 was selected on its ability to elicit high titers of neutralizing antibodies against both SARS-CoV pseudotype virus and live SARS-CoV virus [7, 8], prior to confirmatory testing against SARS-CoV challenge in animal models. It also induced high-level neutralizing antibodies and protective immunity with minimal immunopathology in mice after a homologous virus challenge with SARS-CoV (MA15 strain) [9, 10].

There are several advantages of the CoV RBD candidate antigens and vaccines for purposes of global health:

1. High yield and low cost. The antigens are expressed in *P*. *pastoris*, a low-cost expression platform, which can be produced and scaled at high yields [7, 8]. By deleting an N-linked glycosylated asparagine at the N-1 position of RBD219, both the yield and antigenicity improved. At a 10-liter scale production process, the CoV RBD219-N1 antigen was produced through fermentation at 400 mg/L fermentation supernatant (FS) with purification recovery >50% [7, 8]. A panel of characterization tests indicates that the process is reproducible and robust and that the purified, tag-free RBD219-N1 protein has high purity and a well-defined structure. It is therefore suitable for both pilot scale manufacturing and for transition into process improvements leading to industrial scale manufacturing.
2. Technology transfer. The process is suitable for technology transfer to emerging market vaccine manufacturers (aka DCVMs, developing country vaccine manufacturers) having expertise in fermentation technology (https://www.dcvmn.org/) [11]. The *P*. *pastoris*–derived recombinant protein is currently produced by several DCVMs, including those in Bangladesh, Brazil, Cuba, India, and Indonesia.
3. Shovel ready. The CoV RBD219-N1 antigen was manufactured under cGMP and can be vialed to produce between 20,000 and 200,000 doses, with the possibility of transferring production processes and cell banks to DCVMs for large-scale production sufficient to meet global needs.

Beyond low cost and ease of potential technology transfer to DCVMs, an advantage of employing a recombinant protein subunit vaccine is the long-standing safety record of this class of vaccines, and the fact that this technology has been used for the licensure of two other antiviral vaccines—hepatitis B and human papillomavirus, as well as biologics (e.g., insulin) [11].

## Safety evaluation of a low-cost recombinant vaccine

In addition to their low cost and suitability for use in public immunization programs in low- and middle-income countries, we pursued RBD recombinant protein–based vaccines as a technology to maximize safety relative to other platforms, such as virus vectors that have previously been found to induce immune enhancement. For instance, immune enhancement in children following a formalin-inactivated respiratory syncytial virus (RSV) vaccine was first reported in the 1960s and later shown to occur in laboratory animals with early prototype SARS-CoV vaccines using virus-vectored platforms or inactivated virus constructs [12]. We have recently summarized the major safety concerns of some prototype coronavirus vaccines based on studies conducted in laboratory animals (rodents, ferrets, and nonhuman primates) [12]. They include the following points.

### Avoiding virus-vectored platforms

Some of the earliest SARS-CoV vaccine candidates used vectored-based platforms, and these were associated with immune enhancement or activation. In 2004–2005, scientists at the Public Health Agency of Canada’s National Microbiology Laboratory in Winnipeg, Manitoba (who helped to develop the first successful Ebola vaccine), found that a recombinant modified vaccinia Ankara (rMVA) expressing the S-spike protein resulted in severe liver pathology upon SARS-CoV virus challenge. Similarly, rMVA expressing the S-spike also resulted in lung immunopathology in rhesus macaques, as did other virus-vectored constructs. Lung immunopathology is also linked to whole inactivated viral vaccines. However, it was determined that in many cases eosinophilic pathology is driven by the SARS nucleocapsid (N) protein, although a recent trial in nonhuman primates found that an alum-adjuvanted inactivated SARS-CoV-2 vaccine did not induce immunopathology [13]. Among the major conclusions of these studies was that they may be driven by T helper-17 (Th17) responses linked to interleukin-6 [12, 14], and that aluminum formulations exhibit greatly reduced immunopathology [15].

### Recombinant protein RBD vaccines

Given the history of virus-vector platforms and inactivated vaccines in eliciting eosinophilic immunopathology, our emphasis has been on the evaluation of inexpensive recombinant proteins produced in microbial systems. These are comprised of the CoV RBD219-N1 antigen, encoding amino acids 319–536 (219 AA) of the SARS-CoV S-spike protein [7–10], and now a second, CoV2 RBD antigen, which is also expressed without the N-terminal amino acid. The rationale for selecting the RBD domain of the S protein includes focusing on the key component that binds to the human angiotensin converting enzyme 2 (ACE2) receptor, and removing the known elements of the S protein involved in immune enhancement. Supporting studies summarized elsewhere emphasize how S protein peptides outside of the RBD can induce immune enhancement in non-human primates [12]. Moreover, CoV RBD219-N1 induce high titers of neutralizing antibodies in mice and 100% infection against SARS CoV virus challenge [10]. Alum formulations of CoV RBD 219-N1 do not induce immunopathology [10], a finding consistent with other published studies [13–15].

## Evaluating efficacy

There is evidence to justify advancing the CoV RBD219-N1 antigen as either a homologous vaccine against SARS [7–10] or as a heterologous vaccine against COVID-19 [9]. In parallel, a CoV2 RBD protein candidate is being advanced. Regarding the former, against SARS CoV homologous virus challenge the vaccine formulated on alum exhibits high levels of protective immunity and with evidence of minimal or no immune enhancement [10]. With regards to cross-protection against SARS CoV2, the RBD of the SARS-CoV-2 and CoV RBD219-N1 share significant similarity of amino acid sequence (> 75% identity, >80% similarity) and there is evidence that both viruses use the human ACE2 receptor for cell entry [9]. Further published studies indicate strong antigenic similarities between the SARS-CoV and SARS-CoV-2 RBDs, and the potential for cross protection. For example, serum from a convalescent SARS-CoV patient was shown to neutralize SARS-CoV-2 driven entry [16]. Moreover, new studies by Tai and colleagues find that using pseudotyped SARS-CoV-2, the SARS-CoV RBD blocks the entry of both SARS-CoV and SARS-CoV-2 pseudovirus into human ACE2-expressing 293T cells [17]. Through pseudovirus neutralization activity, it was found that SARS-CoV RBD-specific antisera could neutralize SARS-CoV-2 pseudovirus infection, suggesting that SARS-CoV RBD-specific antibodies can cross-react with SARS-CoV-2 RBD and cross neutralize SARS-CoV-2 pseudovirus infection [17]. Additional studies find that multiple (but not all) neutralizing monoclonal antibodies bind to both RBDs [9, 18, 19].

## Next steps

An international priority is the scale-up and global access of an affordable and safe recombinant vaccine to prevent emerging coronavirus infections, including COVID-19. Our aspirational goal is to protect global populations at risk for this important emerging virus infection. A low-cost recombinant protein antigen expressed in *P*. *pastoris* and formulated on aluminum or other accessible adjuvants represents a highly accessible technology to transfer to low- and middle-income countries. It represents one of several key mechanisms for ensuring that populations across the major affected nations of Africa, Asia, and the Americas will benefit from COVID-19 vaccinations.
